# Recurrence of oral squamous cell carcinoma in surgically treated patients at Khartoum Teaching Dental Hospital retrospective cross-sectional study

**DOI:** 10.1186/s12885-024-12562-6

**Published:** 2024-06-28

**Authors:** Nourelhoda Alim, Mariam Elsheikh, Asim A. Satti, Nafeesa Tabassum, Ahmed M. Suleiman

**Affiliations:** 1 Dar Al Uloom university, Riyadh, Saudi Arabia; 2 Khartoum university, Khartoum, Sudan; 3 Khartoum Teaching Dental Hospital, Khartoum, Sudan

**Keywords:** Oral squamous cell carcinoma, Tumor site, Surgical resection, Radical surgery, Tumor differentiation

## Abstract

**Background:**

In terms of survival rate, recurrent oral squamous cell carcinoma (OSCC) after primary surgery is considered as a poor prognostic indicator.

**Objective:**

This study aims to determine the incidence of OSCC recurrence among patients treated at Khartoum Teaching Dental Hospital (KTDH) and possible risk factors associated with it.

**Methods:**

Records of 303 patients with a history of radical surgery were retrieved from the hospital’s archives, and the histopathological records were retrieved from the archival specimens of Professor Ahmed Suleiman Oral Pathology Laboratory, Faculty of Dentistry, and University of Khartoum.

**Results:**

Advanced stages of OSCC (III, IV) were associated with higher recurrence rates, and the poorly differentiated OSCC was the commonest recurrent type.

**Conclusion:**

The condition of the surgical margin is a significant predictor of OSCC recurrence and tumor stage. The tumor site, the type of surgical resection, and the tumor differentiation were also identified as significant factors influencing the recurrence of OSCC.

## Introduction

Oral cancer is common in males [1]. Physical examination, laboratory testing, and imaging diagnose oral squamous cell carcinoma (OSCC). Over the past years, chemo, radiation, and targeted therapy have improved, but surgery is still the best treatment. Due to local invasion and metastasis, OSCC recurs. OSCC recurrence is a significant prognostic indicator [2]. Recurrence occurs six months after radical tumour resection with safe margins and complete regression [3]. Recurrent rate in OSCC patients is 26% with 40.2%. Mean 5-year overall survival rate [1]. This study examines OSCC recurrence, risk factors, and post-surgical survival rate in OSCC patients.

## Literature review

There are several clinical and demographical variables that influence recurrence of tumor. For instance, age and gender, use of tobacco and alcohol, site of the tumor, stage and grade of the tumor. Age and prognosis are debateable. OSCC in children may reduce aggressiveness and improve prognosis [2]. A study showed no link between age and OSCC recurrence [2], while recurrence mostly observed in males (mean age = 59) [4]. Tobacco-use strongly predicts head and neck squamous cell carcinoma recurrence (HNSCC) [5]. Smokers and drinkers are four times more prone to secondary tumor [2]. In India, buccal mucosa cancer is more common than tongue cancer in the west [5]. Studies reported that tongue cancer contributed to OSCC recurrence after surgery, followed by the floor of the mouth [4, 6, 7]. Numerous studies link OSCC recurrence to tumor (T) stage. OSCC regional recurrence was highly influenced by T and Nodal (N) stages, especially stage III and IV. OSCC recurrence in surgery-only patients is strongly correlated with lymph node metastases [8]. Local, nodal, and distant recurrence patients were mostly in stages III and IV [5]. Stage I-II recurrence individuals have a better prognosis [1].

Treatment variables that influence recurrence include type of surgery, chemo or radiotherapeutic approach, and neck dissection. Marginal mandibulectomy reduces morbidity and treats early-stage cancer [9]. Segmental and marginal resections may avoid postoperative recurrences, SPTs, metastases, and comorbidities [10]. Studies have examined whether OSCC skin flap surgery reduces recurrence and improves survival and suggest that flap repair reduces local tumor recurrence [2]. Neoadjuvant and adjuvant chemotherapy (NAC and AC) may reduce cancer recurrence and improve survival [2, 11–14]. NAC has not improved survival in a study [15–17], whereas NAC found to increase the regional failure in N0 OSCC patients [18]. Stage III and IV radiation treatment failure rates were highest locally and regionally. Unfortunately, local and loco-regional failures are increasing in clinical stages III and IV radiation patients [19]. Moreover, neck dissection is a major predictor of OSCC recurrence in individuals treated with surgery alone. The un-dissected neck has a much higher OSCC recurrence risk [19, 20].

Histopathological variables include, histological grading, perineural invasion, lymphovascular invasion (LVI), and involvement of surgical margins.

Histopathological tumor grading predicts the disease-free and overall survival [6, 21–25]. Poor differentiation predicts OSCC recurrence in surgery-only patients [6, 26], while recurrence rate was shown to be higher in moderately differentiated OSCC [4]. Pathologists call it a perineural invasion when a tumor invades a nerve’s sheath and covering (PNI). Studies have connected PNI to a worse 5-year survival rate, an increased risk of disease recurrence, and regional and distant metastasis [23, 27–30]. A study found that PNI is unrelated to recurrence [2]. While another study reported that OSCC demonstrates that PNI histologically impacts adjuvant treatment decisions and the surgical management of the disease [31].

LVI in cancer cells in an endothelial-lined space (either lymphatic vessels or blood vessels). It is associated with the risk of local recurrences and lymph node metastasis [32]. However, Adel et et al. (2015) reported that LVI does not affect the loco-regional recurrence nor the distant metastasis in patients with OSCC after surgery [33]. Resection of clear surgical margins of a minimum of 5 mm healthy tissues is crucial to prevent local recurrence [34]. It is reported that negative tumor margins are related to a low recurrence rate [2], as 60% recurrence is reported in patients with close resection margins [5].

OSCC recurrence is a major prognostic factor. After surgery, tumor recurrence is associated with a poor prognosis, a decline in quality of life, and an increase in mortality. In spite of excellent surgical resection with negative free margins, neck dissection, and NA therapy, the disease returns in some patients. Literature suggests that the total recurrence rate is 26%, with local, regional, and loco-regional recurrences with 47.3, 35.1, and 10.9%, respectively [1]. Recurrence rates of OSCC is generally high, ranging from 21 to 47.1% [2, 6, 8, 19, 26, 34–37]. Eltohami et al. reported (30%) of patients developed recurrences, and (15%) developed second primary tumours [38]. And 34.9% of oral tongue initial cancers recurred. Recurrence impacts OSCC patients’ 5-year and disease-free survival. 29.1% mean 5-year overall survival percentage following recurrence for advanced stages [1]. It has reported that 30% patients with recurrence survived [6], while local recurrence survival rate is 66.7%.

Local recurrences are caused by leftover cancer cells left after surgery, while SPTs originate from oral mucosal epithelial precursor lesions. The regional recurrence in patients who underwent surgical removal of OSCC alone is the most common recurrence pattern (50%), followed by local (38.9%) and loco-regional (13%) [8, 37]. Kernohan et al. (2010) found that early local recurrence (less than six months after surgery) and late nodal recurrence are worse (6 months or more after the initial surgery). The median recurrence time after surgery is 7.5 months. However, various studies reported 8.5 [7, 37], 14, and 26 months [2, 6] median time for recurrence.

## Methodology

### Study participants

#### Inclusion criteria

The patients meeting the following criteria were selected for the study: (i) preoperative and postoperative pathology confirmed primary OSCC, (ii) OSCC surgery patients at Khartoum Teaching Dental Hospital (KTDH) from 2013 to 2016, (iii) complete clinic-pathologic and follow-up data.

#### Exclusion criteria

Excluded patients were: (i) non-OSCC cancers, (ii) secondary OSCC, (iii) carcinoma with the unknown primary tumor, (iv) incomplete medical records.

### Methods

The Ethics Committee Ministry of Health, Sudan, and the University of Khartoum Research Ethics Board approved this study. Name of the patients were kept confidential and replaced with serial number. Patients were called or questioned during follow-up consultations. A retrospective chart review utilizing a custom-designed data entry sheet recorded demographic and tumor treatment information for the trial. Clinical variables came from KTDH archives, and histopathological variables obtained from Professor Ahmed Suleiman Oral Pathology Laboratory, Faculty of Dentistry, and University of Khartoum archives.

### Sample size and sampling technique

This study did not calculate sample size. Instead, the sample size was determined by the number of patients who fulfilled inclusion and exclusion criteria during recruitment (Convenience sample).

### Data management and statistical analysis

Data was double-checked and transferred to IBMSPSS Inc., Chicago, and version 22 for analysis. Age, gender, cigarette, alcohol, T stage, tumor original location, type of surgery, skin flap, neck dissection, chemo/radiotherapy, histological grading, PNI, surgical margins, and LVI were analyzed using descriptive analysis. Numerical parameters were categorized. The chi-square test compared categorical data. Bivariate Analysis was done. P-value ≤ 0.05 was considered statistically significant and survival rates were analyzed by the Kaplan-Meier method. ICD-10 (International Classification of Diseases) codes determined tumor site.

### Study variables

#### Demographics

1. Patient-related variables.
2. Age.
3. Gender.
4. Habits (snuff dipping, smoking, alcohol).

#### Recurrence variables

1. Date of first diagnosis.
2. Date of surgery.
3. Date of recurrence.
4. Survival after recurrence variables:
5. Last, follow-up date.
6. Date of death.

## Results

### Analysis of demographic variables

The research duration was considered from 2013 to 2016. *N* = 200 males and 103 females. The average age of participants at first diagnosis was 58.9 years (men = 59.2 years, and women 58.2). We observed 130 (42.9%) cases for free safety margins, 118 (38.9%) with involved margins, 46 (15.2%) with close margins, and 9 (3.0%) cases with dysplastic margins. The recurrence rate in patients with free (24.6%), involved (58.5%), close (47.8%), and dysplastic (22.2%) surgical margins was found. Similarly, high recurrence rate (46.2%) was observed with marginal mandibulectomy. The combination of surgery and radiotherapy showed the least recurrence rate (11.8%). Survival rate in patients with recurrence was 4% (2 years), significantly lower than the patients without recurrence (68%). Males were found with more recurrence rates than females.

Among the patient samples, 53.5% were Toombak dippers, while 27.1% were cigarette smokers. Male alcohol usage was modest at 11.6%., and they use Toombak 72.2% more than females (21.4%), (Table 1). Most patients complained of ulceration, edema, and loose teeth. The most common symptoms were a lump (82%), ulcers (73.3%), pain (71.6%), a neck node (48.5%), and teeth mobility (24.8%), (Table 2).

**Table 1:** Frequency of patients’ habits

Habits	Frequency	Percent
Snuff dipping	162	53.5%
Cigarette smoking	82	27.1%
Alcohol	35	11.6%

**Table 2:** Frequency of patients’ complaints

Symptoms	Frequency	Percent
Lump	250	82.5%
Ulcer	222	73.3%
Pain	217	71.6%
Neck node	147	48.5%
Mobile teeth	75	24.8%

### Recurrence variables analysis

The study analysed the recurrence rate with respect to surgical margins. In patients with free surgical margin 24.6% (*n* = 32) recurrence was found. The tumor recurred in 58.5% of patients with involved margins, 47.8% with narrow margins, and 22.2% with dysplastic surgical margins (Table 3).

**Table 3:** Correlation between recurrence and the surgical margin status

Margin status	Recurrence		Non-Recurrence	
Clear	32	24.6%	98	75.4%
Involved	69	58.5%	49	41.5%
Dysplasia	2	22.2%	7	77.8%
Close	22	47.8%	24	52.2%

Chi square test performed, p-value = 0.001, P value is significant

Histopathological assessment showed recurrence in 23.8% and 29.2% patients with positive and negative LVI, respectively. Whereas, recurrence in positive and negative PNI was found to be 28.1% and 26.8%, respectively. Furthermore, 55.6%, 23.1%, and 20.7% recurrence was observed in poorly, moderately and well-differentiated tumors, respectively (Table 4).

**Table 4:** Recurrence in relation to PNI and LVI and tumour differentiation

Variables	Stages	Recurrence		Non-recurrence	
LVI	Positive	5	23.8%	16	76.2%
	Negative	19	29.2%	46	70.8%
	Unknown	8	18.2%	36	81.8%
PNI	Positive	9	28.1%	23	71.9%
	Negative	15	26.8%	41	73.2%
	Unknown	8	19.0%	34	81.0%
Differentiation	Well	18	20.7%	69	79.3%
	Moderate	6	23.1%	20	76.9%
	Poor	5	55.6%	4	44.4%

The site of the primary OSCC significantly affects the recurrence rate of the tumor; the most sites that suffered recurrence were the gum and gingivolabial vestibule (44.1%). The location was identified using codes from ICD-10, (Table 5).

**Table 5:** Tumor site in relation to recurrence

Tumor site	Recurrence		Non-Recurrence	
Lip (C00)	3	21.4%	11	78.6%
Other and unspecified parts of Tongue (C02)	3	10.7%	25	89.3%
Gum (C03)	15	44.1%	19	55.9%
Floor of the mouth(C04)	0	0.0%	1	100.0%
Palate(C05)	3	42.9%	4	57.1%
Other and Unspecified parts of the mouth (C06)	5	29.4%	12	70.6%
Overlapping sites of lip and oral cavity (C14.8)	2	8.7%	21	91.3%
Maxillary sinus(C31.0)	1	16.7%	5	83.3%

The most predominant histological tumor differentiation in the present study is well-differentiated SCC (60.1%), followed by moderately differentiated SCC (27.1%) and poorly differentiated SCC (6.9%). PNI was identified in 76 patients (25.1%), while only 49 patients (16.2%) showed LVI (Table 6). The median time between surgery and recurrence was eight months. Regarding gender preference in relation to recurrence, males (28.7%) tend to have more recurrence than females (16.3%), (Table 7).

**Table 6:** Histological grades of differentiation, PNI, and LVI

Variables	Grades	Frequency	Percent
Tumor Differentiation	Well	182	60.1%
	Moderate	82	27.1%
	Poor	21	6.9%
	Missing	18	5.9%
PNI	Positive	76	25.1%
	Negative	140	46.2%
	Unknown	87	28.7%
LVI	Positive	49	16.2%
	Negative	165	54.5%
	Unknown	89	29.4%

**Table 7:** Distribution of gender among recurrence and non-recurrence groups

Gender	Recurrence		No Recurrence	
Male	25	28.7%	62	71.3%
Female	7	16.3%	36	83.7%

Chi-square test performed, P value = 0.121. The P value is not significant

In this study, T3 and T4 tumors had 22.9 and 27.1% recurrence rates, whereas T1 and T2 had 12.5 and 25.6%. Negative lymph nodes had a 29.2% greater recurrence rate than positive ones (23.8%). Recurrence rises with T stage, but not much (Table 8).

**Table 8:** Recurrence in relation to tumor size, lymph nodes involvement, and TNM stage

Variables	Stages	Recurrence		Non-recurrence	
Tumor size	T1	1	12.5%	7	87.5%
	T2	10	25.6%	29	74.4%
	T3	8	22.9%	27	77.1%
	T4	13	27.1%	35	72.9%
Lymph nodes	Positive	5	23.8%	16	76.2%
	Negative	19	29.2%	46	70.8%
TNM Stage	Stage I	1	12.5%	7	87.5%
	Stage II	7	25.0%	21	75.0%
	Stage III	8	20.5%	31	79.5%
	Stage VI	16	29.1%	39	70.9%

The treatment modalities also affected the recurrence; hence the marginal mandibulectomy had a higher recurrence rate (46.2%) than maxillectomy (33.3%). But the types of neck dissection affect the recurrence significantly, as the modified radical neck dissection (16.4%), and supraomohyoid neck dissection (32.3%) had the least recurrence rate. Level I had the highest rate (40.0%), while those who didn’t have neck dissection have recurrence rate of 33.3% (Table 9).

**Table 9:** **Recurrence in relation to the type of resection and type of neck dissection**

Treatment	Type	Recurrence		Non-Recurrence	
Resection	Segmental	12	22.2%	42	77.8%
	Marginal	6	46.2%	7	53.8%
	Maxillectomy	5	33.3%	10	66.7%
	Soft tissue	9	18.8%	39	81.3%
Neck dissection	Modified Radical	11	16.4%	56	83.6%
	Supraomohyoid	10	32.3%	21	67.7%
	Level I	2	40.0%	3	60.0%
	None	9	33.3%	18	66.7%

In this study, 29.8% patients received the triple treatment developed recurrence, while patients who had surgery + radiotherapy observed with least recurrence rate that is 11.8% (Table 10).

**Table 10:** Recurrence in relation to treatment modalities

Treatment modalities	Recurrence		Non-Recurrence	
Surgery alone	14	23.7%	45	76.3%
Surgery + Chemotherapy	2	28.6%	5	71.4%
Surgery + Radiotherapy	2	11.8%	15	88.2%
Surgery + Chemotherapy + Radiotherapy	14	29.8%	33	70.2%

**Fig. 1:**
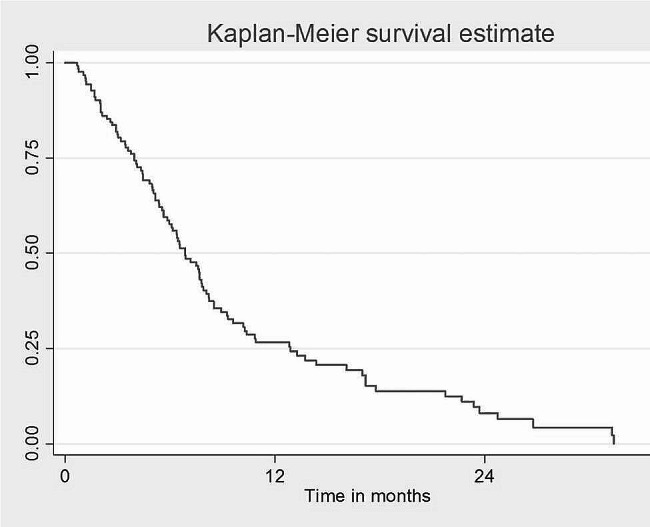
Survival rate of the recurrence group

**Fig. 2:**
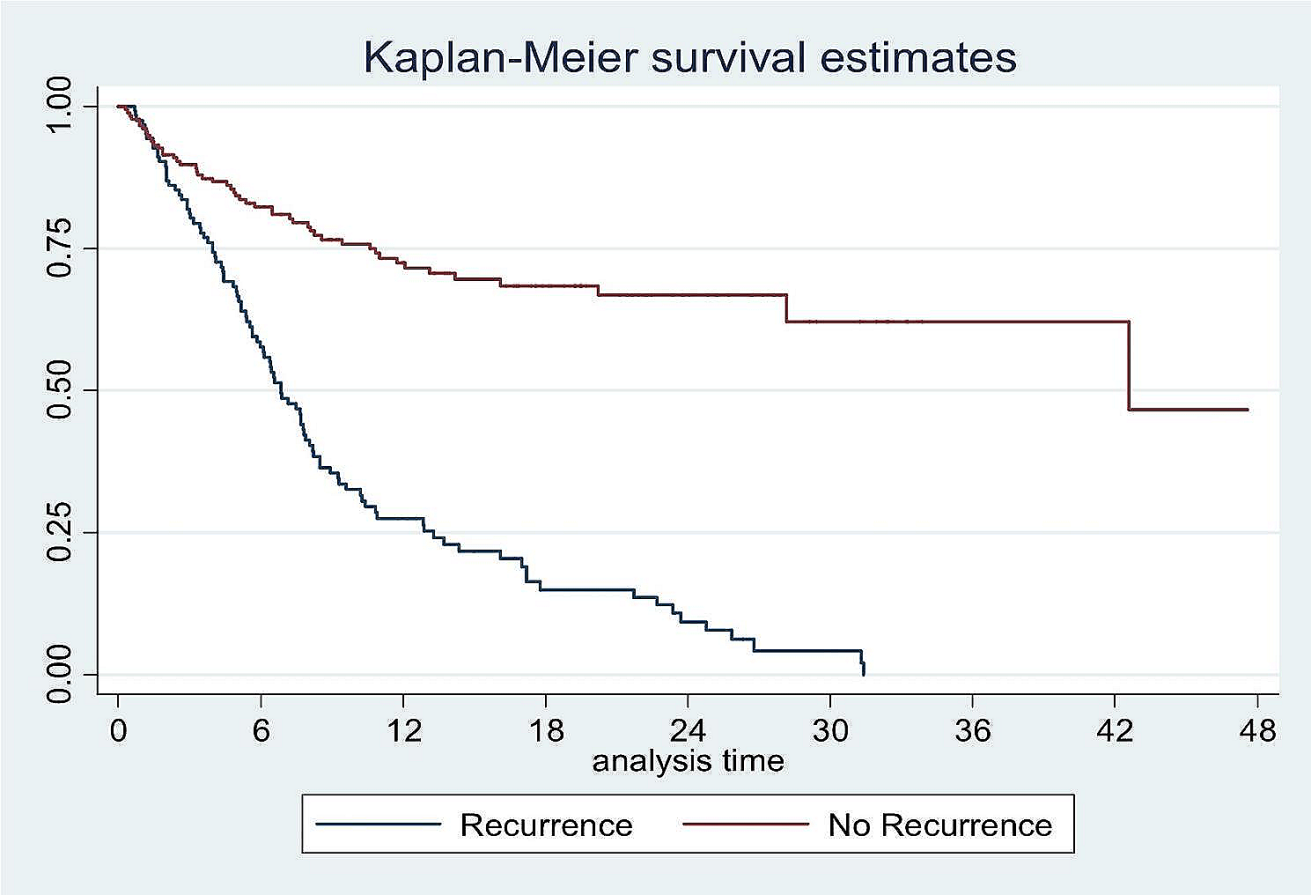
Survival rate of the recurrence group

**Fig. 3:**
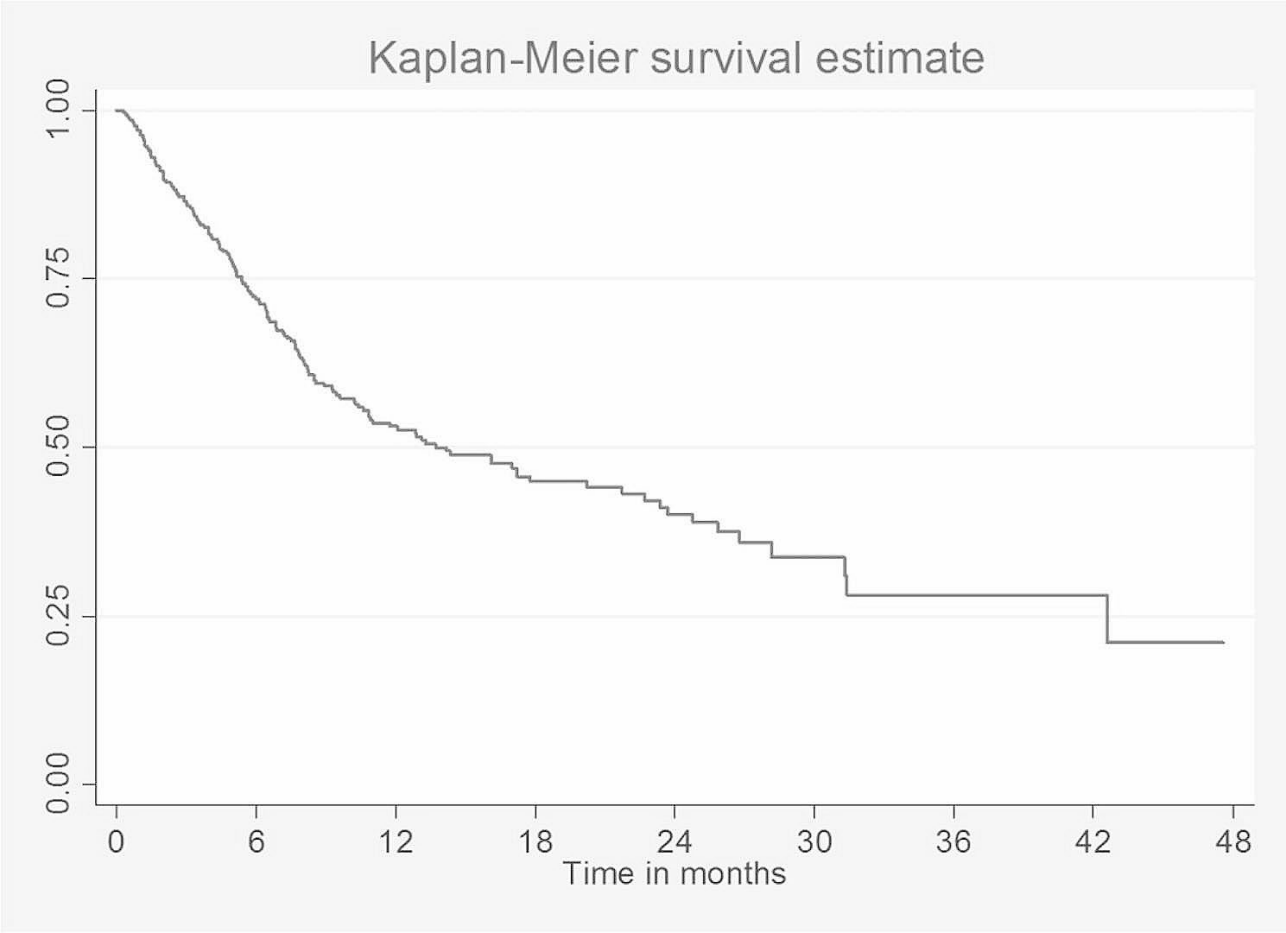
Survival rate of the recurrence group

The mortality rate in all the patients with free surgical margins who developed recurrence was 78.1%, while only 15.6% were alive after recurrence (Table 11). The overall 4 and 2-years survival was 21.1% and 40.0%, respectively, for all OSCC patients who underwent treatment, with its highest records in the first month postoperatively 95% followed by 86.2% in the third month and 71.6% in the sixth month (Fig. 1). In this study, one-year survival was 20% and two-year survival was 4%. All patients died 32 months’ post-recurrence. While in the non-recurrence group, the 2-year survival rate was 68%, and the 4-years survival rate was 45% (Figs. 2 and 3).

**Table 11:** Recurrence in relation to the mortality rate

	Recurrence			Non-Recurrence	
Alive	5	15.6%	68		69.4%
Dead	25	78.1%	26		26.5%
Unknown	2	6.3%	4		4.1%

Chi-square test performed, P value = 0.001, P value is significant

## Discussion

Complete resection of tumors and free margins is the key to good loco-regional control in OSCC. This study examines the recurrence rate of OSCC patients for surgical, clinical and histopathologic parameters, as well as survival after recurrence.

Studies show that older people are more likely to develop OSCC. Llewellyn et al. (2001) found most cases between 50 and 80 years old [39, 40]. Smoking, drinking, and sun exposure make males more likely to get OSCC than women. Males have a 2:1 to 4:1 prevalence rate over women [41–43]. Our Sudanese OSCC patients were usually elderly. Patients with average age of 58.9 years were more likely to be men than women (66%).

Tobacco, alcohol, and betel quid are known OSCC risk factors [44]. In the present study, 53.5% of patients had a history of Toombak dipping, and 27.1% smoked. These numbers are similar to Fang et al. (2013), 61.3% smokers. In this study, 11.6% of patients had a history of alcohol drinking [43].

Many OSCC suffer differently. OSCC lead to ulceration, edema, and tooth loss in patients. The common symptoms found were lump (82.5%), ulcer (73.3%), and mostly pain. In Sudanese investigations, maximum patients had exophytic ulcerated masses on the inner side of the lower lip (gingivolabial) where snuff is dipped [45–47].

The UK Royal College of Pathologists guidelines consider margins of 5 mm or more from the invasive tumor cells as clear margins, 1–5 mm as close margins, and less than 1 mm as involved margins [23]. In the present study, the difference in the recurrence rates between clear, close, and involved excision margins was significant. Clear surgical margins had a 24.6% recurrence rate, similar to Guerra et al. (2003), who reported 21% [48]. The recurrence rate was 47.8% for individuals with close margins and 58.5% for involved margins. Priya et al. (2012) found 30.4% recurrence in close margin, while 41.5% of patients with involved margins did not recur, possibly because most were referred for chemotherapy and radiation or because the postoperative surgical margins evaluation histological diagnosis was incorrect [49]. Struckmeier et al. in Germany reported that Recurrence manifested in 16.63% of the patients, encompassing local recurrence in (77.14% of the patients and distant metastasis in (34.28%). Neck recurrence occurred in only (0.24%) on the contralateral side [50]. Dysplastic cells need time to become malignant, hence close margins (47.8%) had greater recurrence than dysplastic margins (22.2%). Most study shows that diagnosing symptoms are important.

TNM staging and histopathological grading determine outcome. Histological grade predicts recurrence. Poor differentiation had the poorest prognosis and greatest recurrence (55.6%), whereas moderate and well-differentiated had 23.1% and 20.7%, respectively. Eldeeb et al. (2012) found that poorly differentiated tumors had 83.0% greater recurrence rates than moderate and well-differentiated tumors [46]. Priya et al. (2012) observed that histological differentiation did not affect recurrence [49].

Since various cancers affect different bodily parts, the anatomic location of a lesion can predict prognosis [2]. Tongue and floor of mouth SCC has a poor prognosis because to cervical metastases, inaccessibility, and late reporting [7]. In this research, recurrence occurred more often in the gum and gingivo-labial regions (44.1%) following the palate (42.9%). Early observations suggested that mouth floor tumors are harder to cure locally than other oral malignancies. This study found no significant changes because there were few patients with such sub-sites cancers.

PNI in the primary tumor predicts cervical metastasis and loco-regional recurrence, according to Jadhav et al. (2013) [2]. In this study, PNI, LVI, and recurrence were unrelated. In agreement with our findings, Liao et al. (2008) discovered no statistically significant difference between 5-year local control, and overall survival rates of OSCC patients with PNI [34]. In contrast, Brandwein-Gensler et al. (2005) discovered that PNI was a strong independent predictor of local recurrence and overall survival, regardless of tumor margin quality [51].

Gender also affects OSCC recurrence. Wang et al. (2013) found that female patients had fewer recurrence (31.9%) than men (33.7%) [2]. In this study, ladies experienced less recurrence than males (16.3% vs. 28.7%). This may be attributed to the fact that women seek medical treatment earlier and more often than men.

Related to the impact of tumor size on the outcome, it is proven that smaller the tumor size, the better the prognosis. Our study found 38.1% recurrence rate of T1 and T2, while T3 and T4 had a 50% recurrence rate. Similar results were reported by Wang et al. (2013), who found T1 and T2 with 20.3% recurrence rate, while T3 and T4 with 57% [2, 46]. It is also found that negative lymph nodes had a lower recurrence rate (23.3%) than positive ones (48.5%). These findings contradict with present study’s which found that negative lymph nodes had a greater recurrence rate (29.2%) than positive lymph nodes (23.8%). Surgical excision without neck dissection may explain this variation in patients with negative lymph nodes. Clinically negative lymph nodes require prophylactic neck dissection.

TNM staging predicts OSCC recurrence. Stages III and IV had a 49.6% recurrence rate, compared to 37.5% for stages I and II. Sharma et al. (2016) found that most local, nodal, and distant recurrences occurred in stages III and IV [5]. Recurrence with stage I and II primary tumors have a better prognosis [1].

Literature showed that marginal resection technique is not associated with a worse prognosis [48]. This varies from the present study, where marginal mandibulectomy had a greater recurrence rate (46.2%) than segmental (22.2%). The difference may be due to inadequate bone resection in the marginal technique to preserve the mandible’s lower border.

In OSCC patients who have surgery alone, neck dissection type predicts recurrence. Un-dissected necks showed higher OSCC recurrence [19, 20]. In this study, the un-dissected neck had the highest recurrence rate (33.3%), while the modified neck dissection had the lowest (16.4%). Even N0 lymph nodes may contain micro-invasive cancer cells that cause recurrence, requiring neck dissection.

AC improves prognosis and recurrence [2, 11–14]. Compared to surgery + chemotherapy (28.6%) and surgery + radiation (11.8%), the triple therapy combination of surgery, chemotherapy, and radiotherapy had a 29.8% recurrence rate. However, triple-modality patients were sicker.

Several studies indicate cancer survival even after recurrence, such as 5-year survival in 30% cases [6], 2-year survival in 67.6%, and 5-year survival rate in 31.8% [2]. Kernohan et al. (2010) identified an 18% 2-year survival rate for patients who recurred within 3 months postoperatively [7]. Struckmeier et al. reported that the 5-year Overall Survival stood at 58.29%, Patients with early recurrence within ≤ 12 months showed the least favorable prognosis [50]. This study found a 4% (2-years) survival rate and 32-month post-recurrence mortality. In this study, over 80% recurrences occurred within 2 years, validating the current approach of intense and frequent surveillance in the first few years after therapy. Cancer may return despite cancer-free tumor margins. Peripheral cancer cells may not indicate recurrence. Histological type, tumor site, stage, PNI, and depth of invasion increase local recurrence [51].

## Conclusion

Overall, advanced tumor stages III and IV enhance OSCC recurrence. Surgical margin status predicts OSCC recurrence. Modified neck dissection reduced OSCC recurrences in surgically treated individuals. This research may improve treatment strategies for OSCC in Sudan.

## Recommendation

1. OSCC prevention needs improvement.
2. patients should employ intraoperative frozen sections analysis (IFSA) and genetic markers to monitor tumor margin status.
3. Raising OSCC knowledge improves early detection.
4. Patient follow-up must be planned.

### Limitations

While the study adds to previous literature, some limitations must be acknowledged. Participation in this study was limited to a convenient sample of patients, while the sample size was adequate, further research with a larger sample size, including more histopathologic variables and a longer follow up time is recommended.

Table show the histopathological factors used in the study

**Table:** 

Site	Total	percent
Lip	**24**	**7.9%**
Tongue	**44**	**14.5%**
Gum and gingivolabial	**70**	**23.1%**
Floor of the mouth	**6**	**2.0%**
Palate	**36**	**11.9%**
Unspecified	**46**	**15.2%**
Ill-defined sites	**63**	**20.8%**
Maxillary sinus	**14**	**4.6%**
Total	**303**	**100.0%**
	Frequency	Percent
T1	11	3.6
T2	72	23.8
T3	80	26.4
T4	140	46.2
Total	**303**	**100**
	Frequency	Percent
N0	106	35
N1	122	40.3
N2	69	22.8
N3	6	2
Total	**303**	**100**
	Frequency	Percent
Stage I	11	3.6
Stage II	47	15.5
Stage III	90	29.7
Stage IV	155	51.2
Total	**303**	**100**
	Frequency	Percent
free	130	42.9
Involved	118	38.9
dysplasia	9	3
Close	46	15.2
Total	**303**	**100**
Perineural invasion	Frequency	Percent
Positive	76	25.1
Negative	140	46.2
Unknown	87	28.7
Total	303	100
Lymphovascular invasion	Frequency	Percent
Positive	49	16.2
Negative	165	54.5
Unknown	89	29.4
Total	**303**	**100**

## Data Availability

No datasets were generated or analysed during the current study.
